# Supplementary material for: Hot spot prediction in protein-protein interactions by an ensemble system
Source: BMC Syst Biol. 2018 Dec 31;12(Suppl 9):132. doi: 10.1186/s12918-018-0665-8 (PMC6311905; doi:10.1186/s12918-018-0665-8)
Supplement: Supplementary file 2 — All relevant MATLAB code. A simple Matlab implement of our predictor. (ZIP 19,742 kb) [file 12918_2018_665_MOESM2_ESM.zip › Additional file 2/Read ASA/ASA Property/BID/1FCCC.txt]

 NetSurfP - Protein Surface Accessibility and Secondary Structure Predictions

  

|  |  |  |
| --- | --- | --- |
|  |  | NetSurfP - Protein Surface Accessibility and Secondary Structure Predictions  Technical University of Denmark |

  


---

```
# For publication of results, please cite:
# A generic method for assignment of reliability scores applied to solvent accessibility predictions.
# Bent Petersen, Thomas Nordahl Petersen, Pernille Andersen, Morten Nielsen and Claus Lundegaard
# BMC Structural Biology 2009, 9:51 doi:10.1186/1472-6807-9-51
#
# Column 1: Class assignment - B for buried or E for Exposed - Threshold: 25% exposure, but not based on RSA
# Column 2: Amino acid
# Column 3: Sequence name
# Column 4: Amino acid number
# Column 5: Relative Surface Accessibility - RSA
# Column 6: Absolute Surface Accessibility
# Column 7: Z-fit score for RSA prediction
# Column 8: Probability for Alpha-Helix
# Column 9: Probability for Beta-strand
# Column 10: Probability for Coil
E T  1FCCC                  1    0.775 107.548   0.128   0.003   0.003   0.994
E T  1FCCC                  2    0.442  61.333   0.437   0.004   0.514   0.481
B Y  1FCCC                  3    0.162  34.534  -0.426   0.002   0.816   0.182
B K  1FCCC                  4    0.269  55.416   1.089   0.001   0.900   0.099
B L  1FCCC                  5    0.079  14.538  -0.156   0.001   0.959   0.040
B V  1FCCC                  6    0.091  14.048   0.047   0.001   0.959   0.040
B I  1FCCC                  7    0.066  12.136  -0.217   0.001   0.959   0.040
E N  1FCCC                  8    0.347  50.859   0.083   0.003   0.718   0.279
B G  1FCCC                  9    0.207  16.307  -1.481   0.022   0.359   0.619
E K  1FCCC                 10    0.418  85.941  -0.091   0.022   0.552   0.426
E T  1FCCC                 11    0.369  51.236  -1.105   0.023   0.655   0.322
B L  1FCCC                 12    0.194  35.558  -0.284   0.022   0.552   0.426
E K  1FCCC                 13    0.401  82.445  -0.328   0.074   0.484   0.442
B G  1FCCC                 14    0.260  20.446  -1.446   0.135   0.317   0.548
E E  1FCCC                 15    0.451  78.825  -0.351   0.231   0.330   0.439
E T  1FCCC                 16    0.282  39.100  -0.650   0.321   0.252   0.427
B T  1FCCC                 17    0.139  19.335  -0.211   0.502   0.102   0.396
B T  1FCCC                 18    0.249  34.564  -0.261   0.605   0.105   0.290
E E  1FCCC                 19    0.485  84.747   0.446   0.660   0.049   0.291
B A  1FCCC                 20    0.178  19.594  -1.149   0.751   0.050   0.199
B V  1FCCC                 21    0.101  15.508   0.381   0.660   0.049   0.291
E D  1FCCC                 22    0.318  45.853   0.107   0.561   0.047   0.393
B A  1FCCC                 23    0.203  22.349  -0.487   0.879   0.010   0.111
E A  1FCCC                 24    0.429  47.232  -0.223   0.923   0.002   0.076
B T  1FCCC                 25    0.224  30.999   0.295   0.923   0.002   0.076
B A  1FCCC                 26    0.051   5.587  -0.134   0.970   0.001   0.030
E E  1FCCC                 27    0.334  58.367   0.865   0.970   0.001   0.030
E K  1FCCC                 28    0.482  99.230   1.108   0.970   0.001   0.030
B V  1FCCC                 29    0.078  12.050  -0.026   0.970   0.001   0.030
B F  1FCCC                 30    0.038   7.647  -0.501   0.970   0.001   0.030
E K  1FCCC                 31    0.368  75.718   1.529   0.938   0.007   0.055
E Q  1FCCC                 32    0.429  76.655   0.783   0.938   0.007   0.055
B Y  1FCCC                 33    0.105  22.503   0.089   0.879   0.010   0.111
B A  1FCCC                 34    0.109  12.045  -1.146   0.622   0.015   0.363
E N  1FCCC                 35    0.609  89.187   0.050   0.430   0.016   0.555
E D  1FCCC                 36    0.607  87.512  -1.585   0.181   0.016   0.803
E N  1FCCC                 37    0.566  82.877  -1.341   0.058   0.017   0.925
E G  1FCCC                 38    0.349  27.458  -1.596   0.018   0.019   0.964
B V  1FCCC                 39    0.144  22.071  -0.473   0.019   0.141   0.840
E D  1FCCC                 40    0.515  74.269   0.071   0.022   0.359   0.619
B G  1FCCC                 41    0.153  12.017  -0.866   0.004   0.616   0.381
E E  1FCCC                 42    0.396  69.199   0.114   0.018   0.846   0.136
B W  1FCCC                 43    0.141  33.814   0.694   0.011   0.918   0.071
E T  1FCCC                 44    0.329  45.646   0.048   0.018   0.846   0.136
B Y  1FCCC                 45    0.193  41.201   0.346   0.023   0.655   0.322
E D  1FCCC                 46    0.505  72.727  -0.430   0.022   0.359   0.619
E D  1FCCC                 47    0.533  76.849  -0.540   0.020   0.205   0.775
B A  1FCCC                 48    0.255  28.068  -1.038   0.020   0.205   0.775
E T  1FCCC                 49    0.459  63.594  -0.264   0.021   0.451   0.528
E K  1FCCC                 50    0.376  77.281   0.803   0.023   0.655   0.322
E T  1FCCC                 51    0.403  55.841   0.205   0.021   0.756   0.223
B F  1FCCC                 52    0.103  20.632   0.608   0.018   0.846   0.136
E T  1FCCC                 53    0.433  60.002   0.410   0.021   0.756   0.223
B V  1FCCC                 54    0.179  27.435  -1.063   0.023   0.655   0.322
E T  1FCCC                 55    0.490  67.921  -0.683   0.022   0.359   0.619
E E  1FCCC                 56    0.837 146.276   0.155   0.003   0.003   0.994

Explain the output.  Go back.
```

---
